# COVID-19 as an enabler for enhancing online learning and teaching skills for nurse educators at the University of Namibia: Prospects and challenges

**DOI:** 10.4102/hsag.v27i0.1727

**Published:** 2022-02-25

**Authors:** Rakkel N. Shindjabuluka, Daniel O. Ashipala, Gilbert N. Likando

**Affiliations:** 1 Department of General Nursing Science, Faculty of Health Sciences, University of Namibia, Rundu, Namibia; 2 Department of Educational Foundations and Management, Faculty of Education, University of Namibia, Rundu, Namibia

**Keywords:** challenges teaching skills, COVID-19, enablers, nurse educators, online learning, prospects

## Abstract

**Background:**

The coronavirus disease 2019 (COVID-19) pandemic has caused instability in the education system and has compelled higher education institutions (HEIs) to find alternative ways of teaching and learning by making use of the latest online teaching approaches.

**Aim:**

The purpose of the study was to explore how COVID-19 could serve as an enabler for the enhancement of online learning and teaching skills for nurse educators at the University of Namibia with specific emphasis on prospects and challenges.

**Setting:**

Semi-structured interviews were conducted in English at a public nursing education institution located in the northeast of Namibia.

**Methods:**

A qualitative explorative, descriptive and contextual research design was used. Data were collected by means of in-depth semi-structured interviews with 18 nurse educators from the School of Nursing. Data were analysed using thematic analysis. Field notes were simultaneously taken to enrich the data.

**Results:**

The study revealed three themes: nurse educators’ experiences of the use of online learning and teaching skills, COVID-19 as an enabler for enhancing online learning and teaching skills and strategies to sustain online teaching and learning.

**Conclusion:**

Internet technology has generated a surge in demand for web-based teaching and learning. The online learning mode was not effectively utilised during the COVID-19 era because of inadequate technological skills on the part of nurse educators.

**Contribution:**

These findings can be used by universities to equip students and academic staff with skills to adapt to e-learning as the new modus operandi in learning and teaching in the post-COVID-19 era.

## Introduction

The coronavirus disease 2019 (COVID-19) pandemic has been a major disrupter in the learning and teaching milieu and has compelled institutions of higher learning to find alternative ways to address the academic challenges that have accompanied the COVID-19 regulations. Weeden and Cornwell (2020) recognised that the spread of the novel coronavirus (COVID-19) throughout the globe has contributed to significant changes in social contact and the organisation of learning and teaching. In an effort to minimise interpersonal interaction and avoid communal transmission, pandemic interventions called ‘social distancing’ or ‘physical distancing’ have been introduced. Furthermore, the World Health Organization (WHO) (2020) is of the opinion that the transmission of COVID-19 was likely to take place rapidly in dense social networks such as university campuses. The global trend in education systems to respond to the pandemic with emergency e-learning options resulted in the rapid transition from face to face to online learning (Weeden & Cornwell 2020). As one of the best options currently available, the University of Namibia realigned face-to-face learning and teaching to the online environment. The decision to minimise face-to-face learning and teaching and find alternative ways of teaching in all institutions of higher learning in Namibia was taken by the Government of Namibia in line with the guidelines issued by the WHO.

Online learning is Internet-driven education, also called, inter alia, ‘e-learning’. However, this study uses the term ‘online learning’ in reference to e-learning. While online learning has been used in distance education at the University of Namibia (Mpofu et al. 2012), COVID-19 provided the impetus for the University of Namibia to introduce online learning and teaching for contact programmes also. This drastic move was accompanied by the demand for enhanced online skills on the part of nurse educators. Although online learning and teaching programmes were offered in a distance mode prior to COVID-19, the institution was not well prepared and did not have the required online skills to fully shift all programmes offered face to face to online learning. As a result, the institution had to offer training to address educators’ (nurse educators included) inadequate online teaching skills. Adedoyin and Soykan (2020) argued that effective online education involves, inter alia, quality online course design, the planning of instruction and the application of models for the development of instruction and teaching and learning. The absence of course design and an organised model to guide the process of online learning and teaching during COVID-19 falls short of effective online education, rendering it, at best, an emergency response to the COVID-19 crisis.

A study in Kenya that examined the challenges of implementing e-learning at Kenyan universities in general found that insufficient Internet connectivity, lack of e-learning modules, a shortage of computers and insufficient time for online interaction were amongst the challenges nurse educators faced (Tarus, Gichoya & Muumbo 2015). Whilst the study in Kenya hints at possible challenges in implementing e-learning in general, it does not address the prospects of e-learning as an enabler for enhancing the skills of nurse educators at the university level (Mutisya & Makokha 2016). Given the ill-preparedness of many African countries, including Namibia, with regard to information and communication technologies (ICT)-related infrastructure, trained personnel (Simataa 2015) and COVID-19, the move to online learning and teaching had numerous challenges. Despite the challenges, COVID-19 has also brought in new and varied prospects for the higher education sector in Namibia. The School of Nursing has turned to online learning as the only practical strategy for responding to the COVID-19 pandemic. This pedagogical shift had implications for university nurse educators as they were required to possess the requisite skills in instructional technology. In the Namibian context, no study has been undertaken to explore how COVID-19 could serve as an enabler for online learning and teaching skills for university nurse educators’, particularly nurse educators. Therefore, this study aims to ascertain how the pandemic could serve as an enabler for the enhancement of online learning and teaching skills for nurse educators at the University of Namibia using the School of Nursing as a case study.

## Research purpose and objectives

The purpose of the study was to explore nurse educators’ experiences of how COVID-19 served as an enabler for the enhancement of online learning and teaching skills at the University of Namibia with specific emphasis on prospects and challenges.

In order to accomplish the purpose of the study, the following objectives were formulated, namely to:

- Explore and describe how COVID-9 enhanced online learning and teaching at the University of Namibia.
- Explore the challenges nurse educators faced in using online learning and teaching platforms during COVID-19.

## Research methodology and design

The research applied a qualitative approach utilising explorative, descriptive and contextual strategies. The main focus of the research was on identifying, exploring and describing the experiences of the participants. The inquiry process involved the researchers conducting the study in a natural setting and developing a complex, holistic picture, as well as analysing and reporting the detailed views of the participants (Creswell 2014). The goal of qualitative research is to understand rather than explain or predict (Babbie & Mouton 2011). Semi-structured individual interviews were conducted to enable the researchers to understand the experiences of the participants.

### Population, sample and setting

The study took place at one of the University of Namibia satellite campuses located in north-eastern Namibia. The School of Nursing in the Faculty of Health Sciences at this campus only offers a 4-year undergraduate Bachelor of Nursing Science (Clinical) (Honours) degree programme. The university has turned to online blended learning as the only practical strategy for responding to the COVID-19 pandemic. The theoretical knowledge component in each module is offered fully online, whilst the practical knowledge component remains face to face in the clinical learning centre whilst adhering to COVID-19 protocols. The 18 nurse educators in the School of Nursing were included in the sample, with convenient sampling being used to select the research participants.

### Data collection

After approval for the study was granted, the researcher approached the nurse educators to explore their experiences on how their online learning and teaching skills were enhanced during COVID-19. Data in this study were collected in August and September 2020, with one researcher conducting all the interviews under the supervision of the two assigned study leaders. Participants were asked to provide their written consent to participate in the research and were assured that they had a right to withdraw from the study if they so wished although this was not encouraged. Data were collected using an interview guide that was developed based on the research question and the study objectives as well as the literature review. The semi-structured interviews were conducted in accordance with an interview guide, which was formulated to capture nurse educators’ experiences pertaining to online learning and teaching skills during COVID-19 and the prospects and challenges. The interview guide comprised the following sections: (1) ‘Introduction’ requested demographic information from the participants in order to contextualise the research findings in line with this background information; (2) ‘Research purpose and objectives’ asked questions related to nurse educators’ experiences of how COVID-19 has enhanced online learning and teaching skills for nurse educators at the School of Nursing at the University of Namibia; (3) ‘Research methodology and design’ asked questions related to the challenges nurse educators experienced during the facilitation of online learning and teaching and (4) ‘Findings’ asked questions related to whether nurse educators thought there were prospects for change amongst nurse educators to embrace online learning and teaching.

The interviews were conducted by the researcher at a location most convenient for the participants and lasted on average 40–45 min. During the interview, the researcher used an audio recorder whilst also taking notes of the participants’ responses. Data saturation was reached after 15 interviews. As the interviews were conducted face to face, all COVID-19 protocols were observed. For this research, a small-scale study was conducted with three participants from the same sampling unit to establish the relevance and simplicity of the interview questions developed. These participants met the same criteria as the target population. No changes were made to the interview guide as a result of the pilot study, and the results of the pilot interviews were also included in the main study.

### Data analysis

Data from the audio recordings were transcribed verbatim before being analysed using qualitative thematic analysis (Braun et al. 2019). The transcripts and narratives were thematically analysed following Braun six-step method of data analysis that included (1) organising and preparation of data, (2) developing a sense of all the data, (3) coded data following the nine steps of Tesch, (4) identifying and describing themes, (5) representation of the findings and (6) interpretation of the data (Braun et al. 2019). The data were subsequently analysed in order to give meaning (Grove, Burns & Gray 2013). This was done by the researchers and an independent coder experienced in qualitative research. The researchers and the independent coder then held a consensus discussion and agreed on the main themes and sub-themes that emerged. The analysed data were presented using the main research questions and objectives as the key themes.

### Measures to ensure trustworthiness

As this research was conducted qualitatively, the trustworthiness of the study was ensured by using the criteria of Lincoln and Guba (1985), namely credibility, dependability, transferability and confirmability. Accordingly, trustworthiness was addressed as follows:

Credibility in this study was ensured by the researcher who stayed in the field until data saturation was reached to gain an in-depth understanding of the phenomenon. Moreover, credibility was ensured by means of data analysis that truly reflected the data collected for the audit trail. In this study, interviews were performed by the first author who is a postgraduate nursing student in nursing education. This study focused on how COVID-19 could serve as an enabler for the enhancement of online learning and teaching skills for nurse educators. In respect to this relationship, the main dilemma stemmed from the fact that the researcher was a student interviewing nurse educators. The researcher adopted neutrality of the relationship between the participants and the researcher. However, because of power-relation between student and nurse educators, a hierarchical barrier framing the exchange of information will always remain. To ensure that nurse educators provided honest feedback, the researcher developed a trusting relationship that could enable the nurse educators to freely express their feelings about how COVID-19 could serve as an enabler for the enhancement of online learning and teaching skills for nurse educators. To achieve this, the researcher remained open to all possible interpretations of the events and encouraged nurse educators to provide honest feedback. The researcher also openly adopted a non-judgmental stance and as a result nurse educators freely revealed their responses. This unbalanced relationship is systematically taken into account during the research, particularly in the interpretation of data phase. Whenever nurse educators expressed their satisfaction about how COVID-19 could serve as an enabler for the enhancement of online learning and teaching skills, the researcher wondered if their statements were geared to satisfy the researcher. Past experiences with interviews have shown us that because of power relation between student and nurse educators one of the two tends to provide widely positive feedback. Therefore, it has been argued that whatever the respondents say should be treated as valid (Winter 2002). However, this should be subject to caution in asymmetrical relationships such as nurse educators and students. For dependability, the inquiry auditor followed the process and procedures used by the researchers and determined whether they were acceptable. According to Jooste (2010), transferability essentially refers to the generalisation of the data. As Jooste (2010) further stated that in qualitative research, it is often not possible to work with a large and representative sample, therefore the focus in this study was on the quality of information obtained from the participants, situation or event rather than the size of the sample. This was important to guarantee that the findings, conclusions and recommendations were supported by the data, and that there was an internal agreement between the investigators’ interpretation and the actual evidence (Brink, Van der Walt & Van Rensburg 2018). This study used an independent coder to confirm the collected data via the interview guide, which was confirmed by the researchers to make sure that the data were accurate and in line with the information provided by the participants.

### Ethical considerations

After approval from the University of Namibia Research Ethics Committee, ethical clearance to collect data was obtained from the School of Nurse Research Ethics Committee (SoNREC) reference number 04/2020. Participation in this study was voluntary, and participants were free to withdraw during the research although it was not encouraged. Anonymity was explained to the participants with codes being used instead of actual names. Participants were assured of confidentiality because none of the data that were gathered during the research would be linked to any individual participant. Data were stored safely and will be disposed of following the university’s policy on the disposal of research data.

## Findings

Participants in this study were nurse educators who were teaching at the School of Nursing, UNAM, Rundu campus. In addition, participants were under the age of 60 years. A sample consisted of participants from all three departments of the school.

### Participants’ demographical data

The participants were all full-time nurse educators at the University of Namibia. The data in Table 1 indicate the characteristics of the study participants.

**TABLE 1:** Characteristics of participants (*n* = 18).

Characteristics	Total
**Age**	
31–40 years	7
41–50 years	6
51–60 years	5
**Gender**	
Male	5
Female	13
**Marital status**	
Single	2
Married	16
**Rank**	
Junior lecturer	1
Lecturer	15
Senior lecturer	2
**Department**	
Nursing Science	10
Midwifery Science	4
Community Health & Mental Health	4
**Years of experience**	
0–5	15
6–10	3
10+	-

### Presentation and discussion

The themes that emerged from the data analysis are as follows: (1) nurse educators’ experiences of online learning and teaching skills, (2) COVID-19 as an enabler for enhancing the online learning and teaching skills of nurse educators and (3) strategies to sustain online learning and teaching effectiveness (see Table 2).

**TABLE 2:** Summary of findings.

Themes	Sub-themes
Nurse educators’ experiences of online learning and teaching	Positive experiences by nurse educators Challenges faced by nurse educators
COVID-19 as an enabler for enhancing online learning and teaching skills	Improvement of online teaching skills Geographical and time aspects
Strategies to sustain online teaching and learning effectiveness	Training of nurse educators on online teaching and learning Online course designs for instruction and support Communication between nurse educators and students

### Theme 1: Nurse educators’ experiences of online learning and teaching

This theme is a description of the participants’ experiences regarding online teaching and learning during the lockdown resulting from the COVID-19 pandemic. The sub-themes that emerged exposed both positive and negative experiences.

#### Sub-theme 1: Nurse educators’ positive experiences

Participants expressed their satisfaction with online learning and teaching particularly during the COVID-19 pandemic. Participants expressed their satisfaction on how COVID-19 had empowered them to learn new online teaching skills:

‘I think by that I could say that COVID 19 has made me realise that to start engaging in learning online teaching skills faster than it was planned, and as a result, I can now confidently say that I can effectively deliver my teaching through the online platforms.’ (P11, female, 37 years old)

Furthermore, other participants emphasised that online learning created an opportunity to improve their computer skills and gain new knowledge about the Moodle and Panopto learning platforms used at the University of Namibia:

‘I learnt how to use Moodle, I learnt how to use Panopto recording, and I also learnt how to combine both Panopto with WhatsApp when dealing with students. It was an opportunity to learn something new.’ (P10, female, 52 years old)

‘The COVID-19 pandemic really opened my eyes so to speak, when it comes to technology usage because initially we had basically just been doing the traditional teaching, although the online teaching platforms were available in the university, we were not really utilising them, so the COVID-19 pandemic enabled me to know more about online teaching, different teaching platforms, different teaching methods using online.’ (P5, male, 33 years old)

The positive effects of COVID-19 included the fact that it provided an opportunity for institutions to acquire necessary IT infrastructure such as computers and to revamp Internet connectivity and technical equipment (Shalev-Shwartz 2011) to enhance online learning. For many nurse educators who had little exposure to the Internet and with limited online teaching skills, COVID-19 came as a blessing because they gained an opportunity to acquire skills to teach and conduct online assessments.

#### Sub-theme 2: Challenges experienced by nurse educators

Participants reported that online learning and teaching were introduced unexpectedly. Owing to COVID-19 everything was fast tracked, and some of the participants complained about Internet connectivity. Participants also reported their students having similar experiences. Some of the participants’ reflections are highlighted below:

‘It was not really a good experience when the pandemic started, because we never went on any training on how to do the online teaching.’ (P17, male, 39 years old)

‘The negative issues were that we were not prepared enough, we were not trained and the system itself was not ready, it was just trial and error.’ (P4, female, 44 years old)

‘Students were also not ready for online learning since most of them did not have laptops and internet devices but as time went by I saw a lot of improvements, students started getting used to the online learning.’ (P14, female, 52 years old)

Research has found that many African countries including Namibia have inefficient ICT-related infrastructure such as electricity, telecommunications, computers and trained personnel (Leow & Neo 2014). A survey carried out by Biggs (2013) more than eight years ago revealed that Internet connectivity in tertiary institutions in Africa is inadequate, expensive and poorly managed. This situation has not changed much. In the context of the University of Namibia, the above challenges affected some of the participants emotionally and psychologically:

‘… in the beginning things were changing daily and that affected our emotions, and our psychological well-being … we had to adapt accordingly … Furthermore, the institution had issues with the server with internet tripping. At the beginning the server could not accommodate all of us.’ (P1, male, 45 years old)

Participants also revealed how they struggled to use the Moodle and Panopto platforms as well as doing their practical online:

‘The challenges that I was faced were that I was not aware of how to use online platforms such as Moodle and Panopto, especially posting notes on Moodle, how to record, and how to present live sessions with students.’ (P3, female, 52 years old)

### Theme 2: COVID-19 enhanced online learning and teaching skills

This theme focuses on how COVID-19 enhanced online learning and teaching at the University of Namibia, School of Nursing. Based on the data collected, following two sub-themes were generated, namely improvement in online education skills and geographical and time aspects.

#### Sub-theme 1: Improvement in online education skills

The participants highlighted that online learning enhanced student access to the courses taught by qualified nurse educators. This mode of teaching has the advantage of keeping recordings and videos of lectures taught online for students to access in their own time:

‘Yes, its true COVID-19 stimulated and fast tracked online learning process at UNAM.’ (P13, female, 35 years old)

‘COVID-19 boosted my confidence on how to carry out online lectures, I can teach both theory and practical sessions online.’ (P6, female, 43 years old)

‘Online learning and teaching saved me a lot of stresses that include travelling expenses, time and energy as I can just deliver the same information to my students in the comfort of my home.’ (P2, female, 51 years old)

The findings of the study concur with what other researchers have found: that technology is a tool that removes geographical barriers and facilitates learning anytime and anywhere without the presence of nurse educators (Anderson & Magruder 2012; Dag & Gecer 2009; Nguyen 2015). Online learning is an effective mode for delivering education, particularly during the current unprecedented period of the pandemic.

#### Sub-theme 2: Geographical and time aspects

Participants expressed the opinion that online learning was beneficial for both teachers and students. A number of nurse educators commented on their ability to focus more of their attention on the content of the course and less on issues such as parking, traffic and other problems that may arise within a traditional class environment. They further explained that e-learning courses help students get an education without the demands of being located in a specific physical space. The following statements from the participants support these claims:

‘I discovered that we do not need to be at one place to learn.’ (P12, female, 36 years old)

‘It’s more convenient to me as I do not need to drive to and from campus everyday.’ (P7, male, 46 years old)

The above findings support those of Sun and Chen (2016) who asserted that from an economic point of view, a lot of money could be saved by using e-learning, as both teachers and students are not forced to travel to a certain geographical location to access learning. They further believed that teaching that stretches over geographical boundaries would not have been possible without the use of e-learning. Rakap (2010) also argued that time and geographical aspects are two of the key benefits of e-learning.

### Theme 3: Strategies that can be adopted to make online teaching effective

This theme describes what participants mentioned when they were asked to suggest strategies that could make online learning more effective. The sub-themes generated include: training and optimism, online course design, instructions and support and communication.

#### Sub-theme 1: Training of nurse educators on online teaching and learning

A common response from all the participants was that they would like to have more experience and professional training in how to use technological tools for online educational purposes.

‘To make online learning and teaching we need to be trained on how to use different online portals such as: Zoom, Google class, Panopto and Moodle and so on.’ (P16, male, 37 years old)

In the same vein, Meyer and Wilson (2011) recommended that when a learning institution is changing or adopting new ways of teaching, its responsibility is to make sure that it provides resources that are needed by both students and teachers, including training their personnel in how to use this modern way of teaching. Anderson and Magruder (2012) stressed that teachers and professors must be continuously educated on how to use technology in education for effective instruction and assessment to take place.

#### Sub-theme 2: Online course design, instructions and support

Participants required support in designing, developing, evaluating and marking assignments for online modules. This strategy would help students appreciate online learning and understand it better:

‘We still need more time to design all modules to make online modules and courses. This is difficult to do without the assistance of the university.’ (P8, female, 33 years old)

‘Designing soft copy notes, assignments, tests, shooting videos and recording audios has facilitated adoption of online mode of learning.’ (P15, female, 48 years old)

According to Van Bruggen (2005), students carrying out online learning want courses that are well designed, as well as clarity on assignments and feedback that is consistent and timely. Hennessy, Harrison and Wamakote (2010) advised that instructors who design courses should help nurse educators to adapt to new technologies.

#### Sub-theme 3: Communication between nurse educators and students

Participants raised concerns with regard to different ways of communicating during online learning and teaching:

‘I think capturing online lesson videos helps to see the gestures of the student and assess whether they understand or not.’ (P10, female, 54 years old)

‘We can facilitate communication among students by giving group work that could be done and assessed online.’ (P18, female, 50 years old)

‘As a nurse educator I think we need consistent feedback from our students. This feedback helps a nurse educators to improve teaching skills online.’ (P13, female, 40 years old)

According to Biggs (2013), there is a multitude of options for students to work collaboratively and cooperatively with other learners and/or nurse educators. These include: online live debates, reflective journal entries, peer reviews, discussion boards and video or audio teleconferencing (Meyer & Wilson 2011). At the University of Namibia, the Panopto online platform provides such opportunities and experiences.

### Strengths, limitations and areas for further research

The strength of this study lies in the fact that nurse educators’ views were considered from their perspective. This study focused on the experiences of nurse educators at the University of Namibia located in the north-east of Namibia. Consequently, their experiences of how COVID-19 could serve as an enabler for the enhancement of online learning and teaching skills for nurse educators at the University of Namibia. Although the findings cannot be generalised, there are still areas that require further studies; for example, further research is required on a skills audit for nurse educators to enable the institution to mount relevant interventions.

## Recommendations

The recommendations are based on the literature and the findings of this study. Accordingly, the researcher recommends the following: (1) online skills training should be provided to nurse educators in advance, (2) ICT staff should maintain the IT infrastructure such that it will effectively support online learning and teaching and (3) nurse educators should be provided with new well-functioning computers with the latest software to support their online teaching.

## Conclusions

The purpose of the study was to explore and describe nurse educators’ experiences of how COVID-19 could serve as an enabler for the enhancement of online learning and teaching skills for nurse educators at the University of Namibia, with specific emphasis on prospects and challenges. The findings of this study revealed that nurse educators had both positive and negative experiences regarding the use of online teaching and learning skills during the lockdown resulting from the COVID-19 pandemic. The positive aspects were that online learning created an opportunity for them to improve their computer skills, as well as gain new knowledge and skills relating to the Moodle and Panopto online learning platforms. The negative aspects included poor Internet connectivity that affected online learning and teaching and inadequate knowledge on the part of nurse educators on how to use the online platforms. There is consequently an imperative need for training in and the acquisition of technological skills for conducting online teaching, learning and assessment to optimise online experiences.

The findings also revealed that there is a need for strategies to guide the implementation of online learning during COVID-19. Apart from enhancing training and communication as strategies, the findings revealed that there is a critical need to design a model for guiding the planning and development of online learning and teaching instruction. This model would assist institutions of higher learning on how matters relating to the quality assurance of assessment could be addressed given the complexity of online instruction. The findings of the study have significant benefits for various stakeholders. For universities, the online learning will equip students and academic staff with skills to adapt to e-learning as the new modus operandi in learning and teaching in the post-COVID-19 era. The Ministry of Higher Education, Training and Innovation will accordingly be sensitised to the need to ensure that all institutions of higher learning in Namibia embrace online learning and teaching effectively and develop policies to support their curricula implementation post-COVID-19. Quality assurance regulating bodies such as the Namibia Qualification Authority (NQA) and the National Council of Higher Education (NCHE) will gain insight on how to maintain the high integrity levels and educational standards of qualifications conducted via e-learning. Finally, other stakeholders in the education sector such as student bodies and unions will understand the need to recognise and support the implementation of online platforms in learning and teaching, post-COVID-19.
